# Tough Ionogels: Synthesis, Toughening Mechanisms, and Mechanical Properties—A Perspective

**DOI:** 10.1021/jacsau.2c00489

**Published:** 2022-11-28

**Authors:** Meixiang Wang, Jian Hu, Michael D. Dickey

**Affiliations:** † Department of Chemical and Biomolecular Engineering, North Carolina State University, 911 Partners Way, Raleigh, North Carolina 27695, United States; ‡ State Key Lab for Strength and Vibration of Mechanical Structures, Department of Engineering Mechanics, Xi’an Jiaotong University, Xi’an 710049, China

**Keywords:** ionogels, tough, phase separation, solvent exchange, double network, noncovalent cross-links

## Abstract

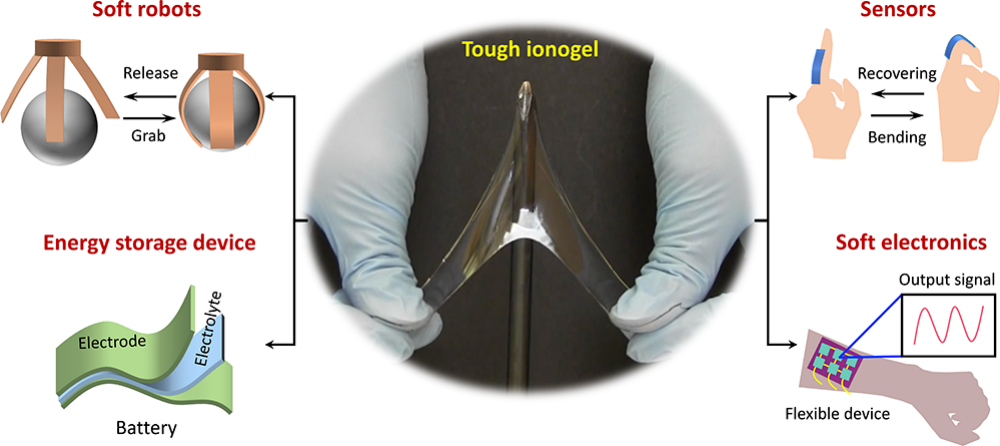

Polymeric ionogels are polymer networks swollen with ionic liquids (*i*.*e*., salts with low melting points). Ionogels are interesting due to their unique features such as nonvolatility, high thermal and electrochemical stability, excellent ionic conductivity, and nonflammability. These properties enable applications such as unconventional electronics, energy storage devices (*i.e.*, batteries and supercapacitors), sensors and actuators. However, the poor mechanical performance of ionogels (*e*.*g*., fracture strength < 1 MPa, modulus < 0.1 MPa, and toughness < 1000 J m^–2^) have limited their use, thus motivating the need for tough ionogels. This Perspective summarizes recent advances toward tough ionogels by highlighting synthetic methods and toughening mechanisms. Opportunities and promising applications of tough ionogels are also discussed.

## 1 Introduction

Polymer networks swollen with solvents are often called gels.^1−4^ Depending on the solvents, gels could be classified into hydrogels (water as solvent), organogels (organic solvents), and ionogels (ionic liquids (ILs) as solvents).^5−8^ Among them, ionogels have aroused much attention due to their unique properties including ionic conductivity, nonvolatility (*i*.*e*., they do not evaporate), and high thermal and electrochemical stability.^9−11^ Ionogels inherit these outstanding features from the ILs that solvate the polymer network. Although we focus here on polymeric ionogels, we note that there are also inorganic ionogels. These ionogels involve matrices, such as metal oxides (*e*.*g*., TiO_2_) or metal organic frameworks (MOFs), swollen with ILs.

**Figure 1:**
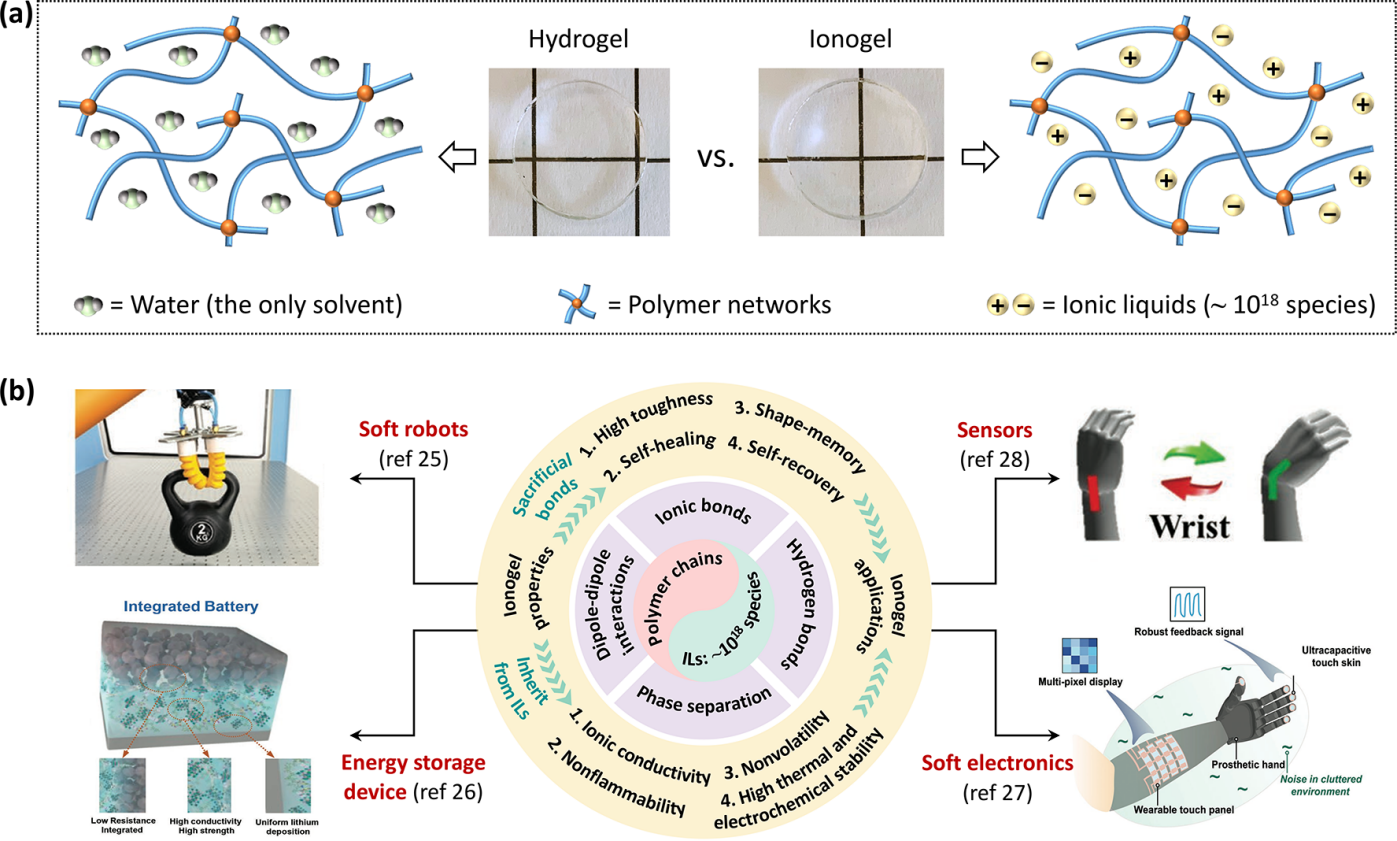
Properties and applications of ionogels. (a) Schematic depicting the structures of a hydrogel and ionogel. (b) Ionogels consist of polymers swollen with ionic liquids (ILs), of which there are an estimated ∼10^18^ ILs possible. The ILs can interact with the polymer in ways defined by the purple concentric band. The tan colored outer band shows properties that lead to applications, of which several are highlighted on the periphery. Applications in (b) were reproduced with permission from ref (25) (Copyright 2021 Wiley-VCH), ref (26) (Copyright 2021 Wiley-VCH), ref (27) (Copyright 2021 Wiley-VCH), and ref (28) (Copyright 2022 Wiley-VCH).

ILs are molten salts consisting of cations and anions, which are liquid at low temperature (generally < 100 °C).^12−14^ Most familiar salts, such as sodium chloride, are solids at room temperature. Relative to conventional salts, ILs have bulky side groups that frustrate crystallization and therefore lower the melting point. Importantly, there are many different possible synthetic ILs with tunable properties such as biocompatibility, ionic conductivity, and hydrophilicity. Approximately 10^18^ ILs could form by combining different cations and anions.^9,12,15^ Therefore, ILs are very different in physicochemical properties, resulting in a wide variety of ionogels (Figure 1a). This further differentiates ionogels from hydrogels, since water is the only solvent for hydrogels.^2,12,16,17^ Taken in sum, there are many attractive features of using ILs to solvate gels instead of water, yet ILs are not without drawback: currently ILs are more expensive and viscous than water.

Ionogels can be formed by swelling a polymer network with ILs or by polymerizing monomers in an IL solvent. As chemically diverse and charged solvents, ILs can have many physical (*i*.*e*., noncovalent) interactions with polymer networks. For example, electrostatic, dipole–dipole, ionic, and hydrogen bonds can form between polymer chains and ILs if they have good compatibility (Figure 1b).^7,12,13,18^ If the compatibility is poor, the polymer chains can phase separate by excluding ILs.^2,18,19^ Additionally, the topology (*i*.*e*., entanglement) of polymer networks also greatly affects ionogel mechanical properties. Topological ionogels can be created by forming entanglements between polymer chains or between polymer chains and physical cross-linkers (*e*.*g*., microgels or MOFs crystal).^20,21^ The noncovalent bonds, phase behavior, and entangled topological architecture can dissipate energy while deforming the ionogel. Therefore, the ionogels may be toughened using chemistry and judicious choice of ILs. In cases in which the interactions are reversible (that is, they can reform after breaking), it is possible to create ionogels with self-healing, shape-memory, or self-recovery properties.^22−24^ Combining these promising properties with the excellent features of ILs makes ionogels attractive materials for ionotronics (an analog to electronics in which the function comes from the movement of ions rather than electrons) and extreme environments (*i*.*e*., high vacuum, low or high temperature). Furthermore, ionogels also show great potential in sensors, energy storage devices, soft robotics, and flexible electronics (Figure 1b).^25−30^

Despite their promise, existing ionogels mostly have poor mechanical properties (fracture strength: < 1 MPa, fracture energy: < 1000 J m^–2^), which limits their applications.^23,31,32^ Polymer networks can be toughened by designing energy dissipation mechanisms into the network.^3,33−35^ Significant work has been done to toughen hydrogels. For example, the fracture strength and fracture energy of a toughened hydrogel are ∼10 MPa and ∼10 000 J m^–2^.^34^ A popular toughening strategy relies on introducing sacrificial bonds that break to dissipate energy during deformation.^35−37^ Several synthesis strategies have been developed to introduce sacrificial bonds into ionogel networks, as reviewed herein.^22,38−40^

This Perspective seeks to highlight existing tough ionogels and provide a set of rational principles for designing tough ionogels based on energy dissipation toughening. A brief introduction of tough ionogels and a summary of their mechanical properties are first discussed. Then we systematically discuss recent progress on tough ionogels from synthetic strategies and toughening mechanisms to mechanical properties. Lastly, future opportunities and possible applications of tough ionogels are also discussed. Importantly, this is a relatively “young” field; there are many opportunities to harness the power of synthetic chemistry combined with the chemical diversity of ILs to create new materials (or existing materials in simpler ways). Overall, the Perspective introduces the latest advances in tough ionogels and establishes guidelines for the design of next-generation tough ionogels.

## 2 Mechanical Properties of Tough Ionogels

**Figure 2:**
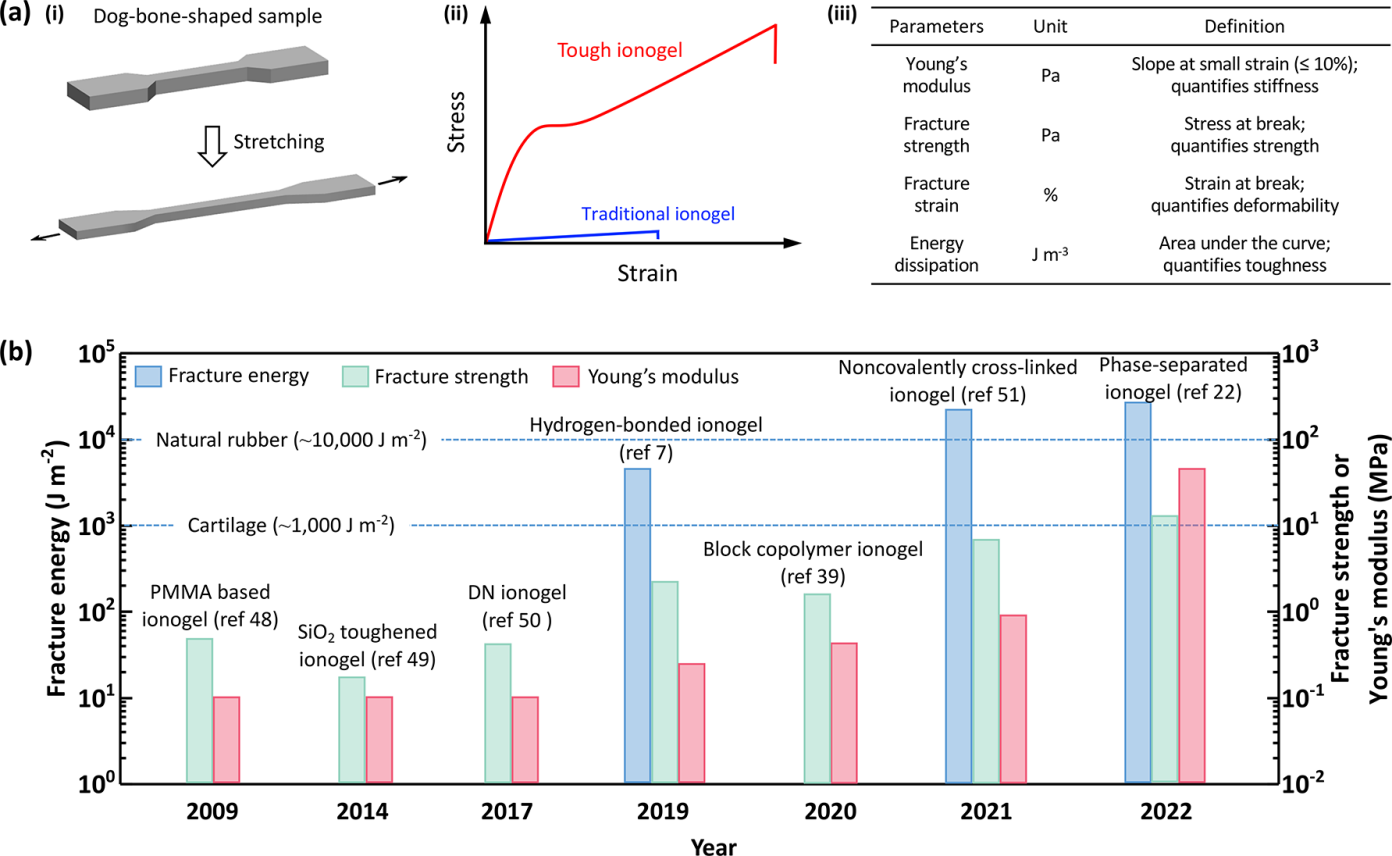
Mechanical properties of tough ionogels. (a) The tensile measurement (i) and stress–strain curves (ii) of ionogels and the mechanical properties obtained from the stress–strain curve (iii). (b) Summary of the mechanical properties of various ionogels (*i*.*e*., PMMA based ionogel,^48^ SiO_2_ toughened ionogel,^49^ double-network (DN) ionogel,^50^ hydrogen-bonded ionogel,^7^ block copolymer ionogel,^39^ noncovalently cross-linked ionogel,^51^ and phase-separated ionogel^22^) in terms of fracture energy, fracture strength, and Young’s modulus. For some tough ionogels, they rival or exceed the fracture energy of cartilage and natural rubber.

A tough ionogel should sustain high stress and high strain (Figure 2a).^33^ Yet, most ionogels reported to date generally fail at low stress and small strain.^41−43^ These parameters can be obtained from the tensile stress–strain (s-s) curves that evaluate material properties. For instance, Young’s modulus is calculated from the slope of the s-s curve at low strain (≤ 10%), which describes the material stiffness. Fracture strength and fracture strain are the stress and strain at break, which presents the strength and deformability of a material, respectively. Energy dissipation is the area under the s-s curve indicating the energy dissipated per unit volume to estimate the material toughness.^33,44^ Additionally, fracture energy is another widely adopted parameter to quantify the toughness of a material. A material with higher fracture energy tends to be able to sustain higher levels of stress and strain, and therefore has a higher energy dissipation. Fracture energy quantifies the ease in which cracks propagate in a material and can be measured by a pure-shear test.^3,44^

Figure 2b summarizes the recent development of ionogel mechanical properties in terms of fracture energy, fracture strength, and Young’s modulus. To the best of our knowledge, an ionogel was first reported in 1993.^2,45^ Afterward, efforts focused to understand this new class of materials by studying the thermodynamic properties (*i*.*e*., phase behavior and solubility) of polymers in ILs.^19,41,46,47^ The initial applications of ionogels were mainly based on their ionic conductivity. To expand their applications, the mechanical properties of ionogels have gradually attracted attention since 2009.^48^Figure 2b shows the evolution of mechanical properties of ionogels, which have been significantly enhanced. For example, the fracture energy (from ∼4700 to ∼24 000 J m^–2^), fracture strength (from ∼0.2 to 13 MPa), and Young’s modulus (from ∼0.1 to ∼50 MPa) have improved by several orders of magnitude.^7,22,39,48−51^ Notably, some toughened ionogels are comparable to or outperform tough gels found in nature, such as cartilage and natural rubber.^3,52,53^

In addition to improved mechanical properties, ionogels with multiple functions (such as self-healing) have also emerged, which can be achieved by introducing reversible bonds into the polymer network. For instance, tough ionogels with physical networks were created by forming interpenetrating entanglement between the polymer chains and fillers (*i*.*e*., covalently cross-linked microgel spheres or MOFs crystal) and electrostatic interactions and hydrogen bonds between polymer networks and ILs.^20,21^ These reversible interactions enable self-healing and recyclable properties to ionogels. Furthermore, the fillers effectively eliminate stress concentrations in the network making the ionogels notch-insensitive and imparting high toughness and good fatigue resistance.

## 3 Toughening Mechanisms

Ionogels can be toughened by introducing sacrificial bonds into polymer networks to dissipate energy. In this section, we summarize the strategies to achieve these sacrificial bonds, such as solvent exchange and phase separation. Then we classify ionogels according to the toughening mechanisms: double-network (DN), noncovalent cross-links, and phase separation. We also discuss the resulting mechanical properties.

**Figure 3:**
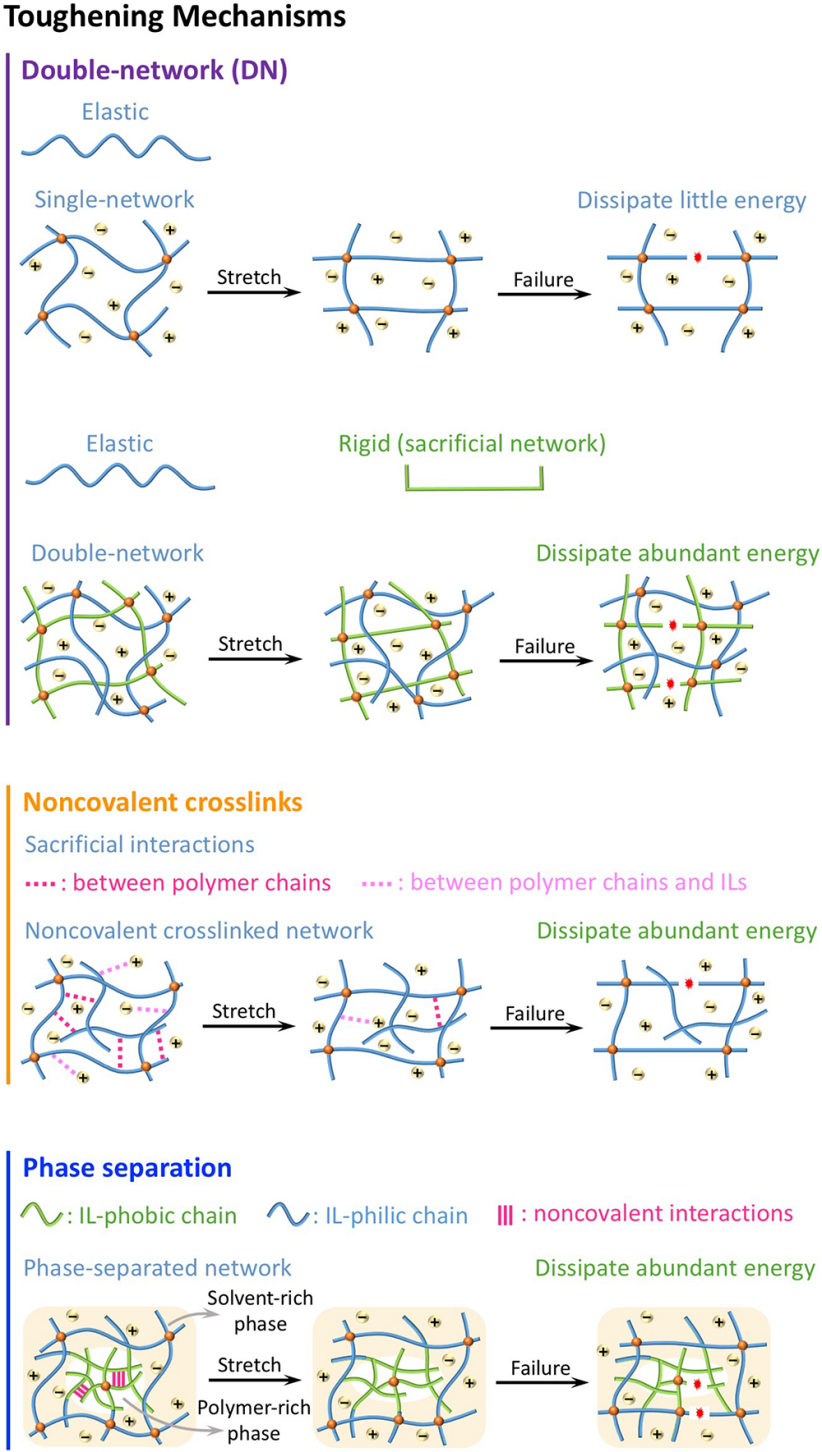
Schematics illustrating toughening mechanisms in ionogels. For DN ionogels, rupturing the rigid polymer dissipates energy while the elastic portions maintain the network. For noncovalent cross-linked ionogels, breaking noncovalent interactions dissipates energy. The noncovalent interactions form both between the polymer chains and between the ILs and polymer chains. In phase-separated ionogels, IL-phobic chains form phase-separated domains that bond via noncovalent interactions, while IL-philic chains remain highly solvated. Disrupting the noncovalent interactions in the phase-separated domains dissipates energy, thereby toughening the ionogels.

Figure 3 illustrates common toughening mechanisms of ionogels. First, consider DN ionogels.^50,54,55^ In single-network ionogels, the polymer network is elastic and cannot effectively dissipate energy via elongating polymer chains, resulting in weak ionogels.^32,56^ In contrast, in DN ionogels, an elastic network is combined with a brittle network. The elastic network is soft, while the brittle network is rigid. When stretching, the rigid network will fail first to dissipate energy and thus toughen the ionogels.^54,55,57^ Second, noncovalent interactions (such as ionic bonds, hydrogen bonds, electrostatic interactions) are usually used as sacrificial (or weak) bonds to improve the mechanical properties of ionogels.^23,38,58−60^ Note that ILs are charged solvents and therefore can interact with polymers in unique ways relative to other solvents such as water. Noncovalent bonds can also form between polymer chains.^38,58,61^ Such noncovalent bonds dissipate energy during deformation by rupturing/reforming these bonds, thereby strengthening the ionogels. Finally, the mechanical properties of ionogels can be enhanced by decreasing the solubility of polymers in ILs.^22,38^ The polymers that have poor compatibility (IL-phobic) with ILs will aggregate to form phase-separated domains that act as sacrificial/reversible interactions, while the polymers with good compatibility (IL-philic) are highly solvated in ILs to form uniform networks.^22,62^ Therefore, ionogels containing these two polymers will exhibit two phases: polymer-rich phase (rigid domain) and solvent-rich phase (soft domain) (Figure 3). In the polymer-rich phase, noncovalent interactions are generally formed due to the close distance between the polymer chains. When loading the phase-separated ionogels, noncovalent interactions in the polymer-rich phase break to dissipate enormous amounts of energy and thereby toughen the gels. The detailed descriptions and examples of these toughening principles are further discussed in the following sections.

### 3.1 Double-Network (DN)

#### 3.1.1 Polymer Based DN

**Figure 4:**
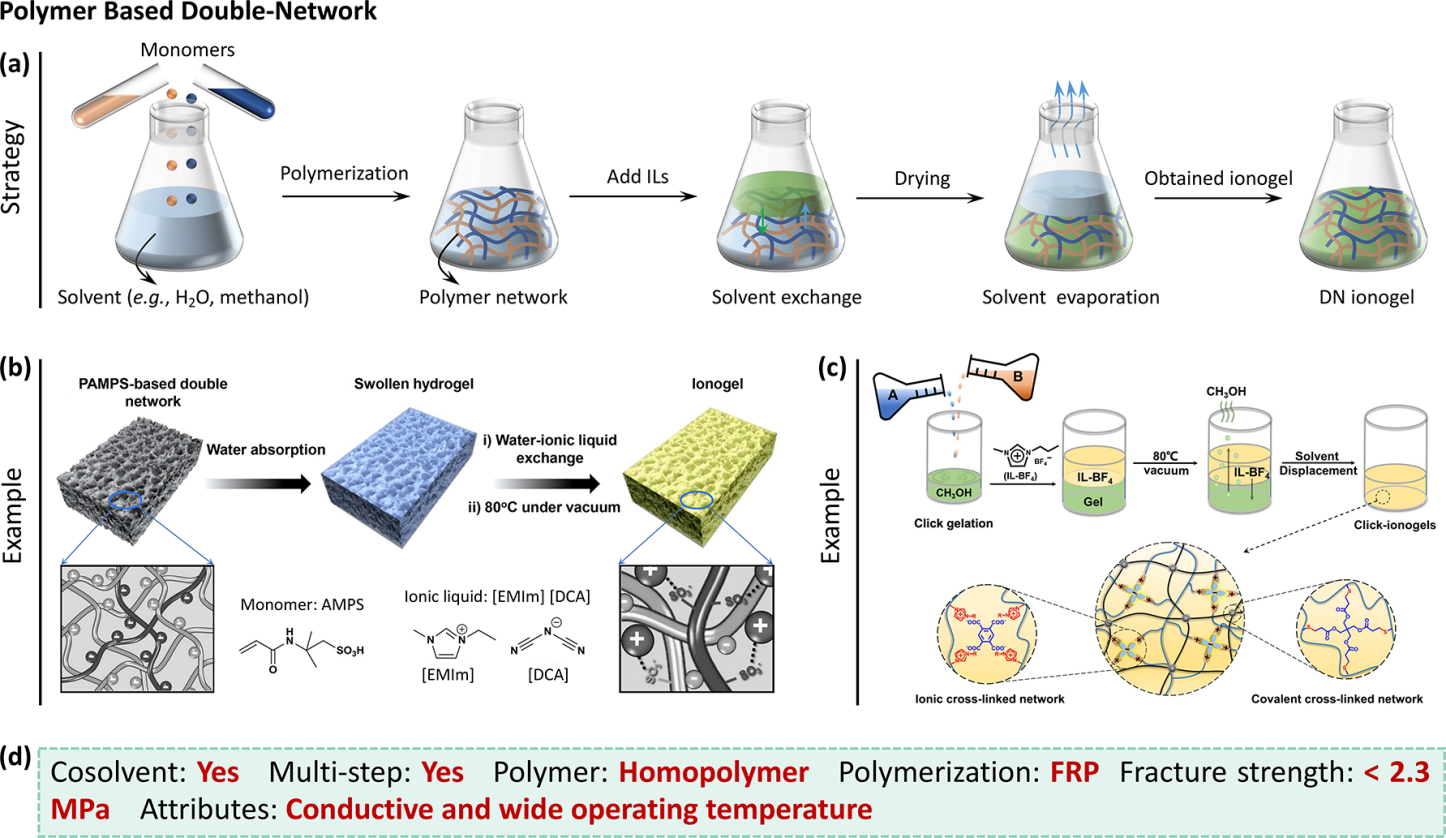
Polymer based double-network (DN) ionogels. (a) Synthetic process of solvent exchange strategy. This strategy is usually laborious and costly due to the complicated process. (b) Fabrication of PAMPS DN ionogel (Reproduced with permission from ref (50). Copyright 2017 Wiley-VCH) and (c) click-based DN ionogel (Reproduced with permission from ref (40). Copyright 2019 AAAS. This image adapted from ref (40) is licensed under CC BY 4.0.)^40,50^ (d) Synthesis features and properties of DN ionogels (FRP = free-radical polymerization).

Introducing a second network into another polymer network is a typical strategy to improve the mechanical performance of gels.^60,63,64^ The resulting gel is called a DN gel. For example, a DN hydrogel reported by Gong *et al.* is believed to be the first tough hydrogel with mechanical properties comparable to natural rubber and cartilage.^65^ These principles can also be used to toughen ionogels. For instance, researchers created DN ionogels using multiple steps such as solvent exchange. Here, solvent exchange refers to the process of forming polymer in one solvent and then later switching the solvent to IL. Typically, solvent exchange involves first dissolving monomers into an organic solvent, inducing free-radical polymerization (FRP) in that solvent, adding IL, and then evaporating the organic solvent, leaving behind polymer in IL (Figure 4a). In principle it would be simpler to polymerize directly in ILs in a single step. Yet, the more laborious process of solvent exchange (from common solvents to ILs) is often used due to difficulties picking ILs that provide appropriate solubility of monomers and polymers to control phase behavior. For example, researchers used a three-step method to prepare a poly(2-acrylamido-2-methyl-1-propanesulfonic acid) (PAMPS) DN hydrogel and then soaked it in the IL to exchange water for IL (Figure 4b).^50^ Finally, residual moisture was removed under vacuum at 80 °C to obtain the PAMPS DN ionogel. Due to the significant energy dissipation of the brittle network, this DN ionogel showed a high compressive strength of 7.7 MPa at 92% strain, which clearly outperformed most conventional ionogels (∼0.1 MPa).^23,32^ Although the compressive properties of the PAMPS DN ionogel were markedly enhanced to some extent, the tensile properties were still poor (*e*.*g*., fracture strength: 0.4 MPa, fracture strain: 158%). Moreover, the multistep synthetic process of PAMPS DN ionogel is laborious and costly.

Thereafter, a click-based DN ionogel composed of poly(ethylene glycol) diacrylate and poly(1-butyl-3-vinyl imidazolium tetrafluoroborate) networks was reported via solvent-exchanging methanol for IL of 1-butyl-3-methylimidazolium tetrafluoroborate.^40^ The mechanical properties of the click-based DN ionogel are significantly improved compared to the aforementioned PAMPS DN ionogel.^50^ As shown in Figure 4c, the two networks in the click-based DN ionogel are, respectively, ionically and covalently cross-linked. When deforming this DN ionogel, the ionic bonds serve as sacrificial bonds to dissipate energy and thus toughen the ionogel, while the covalently cross-linked network maintains the network, resulting in high stretchability. This click-based DN ionogel gave a fracture strength of 2.28 MPa with a fracture strain of ∼1400% and a compressive strength as high as 23.7 MPa at a strain of 92%. These mechanical properties are comparable to biological tissues, such as cartilage. However, the synthesis of the click-based DN ionogel is not straightforward and usually takes several days.^40^

The features and properties of the synthetic strategies of these DN ionogels have been summarized in Figure 4d. These approaches involve multiple steps. The resulting ionogels are homopolymer networks fabricated from FRP. Compared with traditional ionogels, these ionogels exhibit improved mechanical properties, excellent ionic conductivity, and a wide range of operating temperature (*e*.*g*., from −40 to 120 °C).^40^ New approaches are needed to improve the tensile properties while simplifying the fabrication process, as these factors play a key role in practical applications.

#### 3.1.2 Polymer and SiO_2_ Hybrid DN

**Figure 5:**
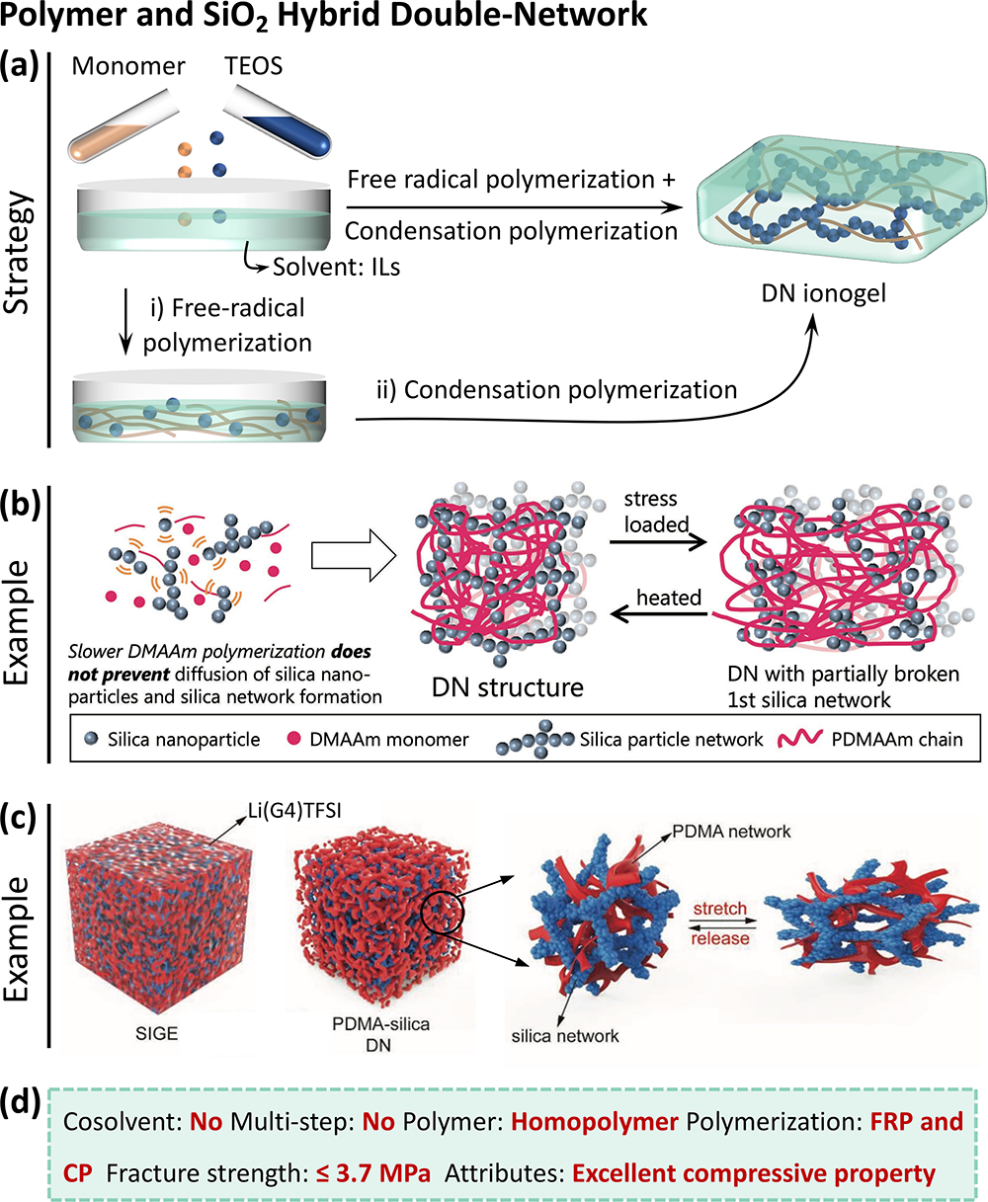
Polymer and SiO_2_ hybrid DN ionogels. (a) Synthetic process of DN ionogels by one-step and two-step approaches. (b) Two-step method: TEOS polycondensation as the first network, followed by the free-radical polymerization of DMAAm monomer as the second network.^54^ Reproduced with permission from ref (54). Copyright 2017 Wiley-VCH. (c) The process in (b) was simplified into one step through simultaneous TEOS polycondensation and DMAAm polymerization.^55^ Reproduced with permission from ref (55), Copyright 2019 Wiley-VCH. (d) Summary of the synthetic approaches and resultant properties of the DN ionogels.

To address the above issues, some simple approaches to achieve tough DN ionogels have been developed. Among them, introducing a silica network has aroused much attention due to its ease of fabrication.^54,55^ Moreover, silica can notably improve the mechanical properties by forming noncovalent bonds between themselves or with the polymer networks to dissipate energy.^5,66^ For example, an inorganic–organic DN ionogel composed of a silica network and a poly(*N*,*N*′-dimethylacrylamide) (PDMAAm) network was created (Figure 5b).^54^ This DN ionogel was prepared by condensation polymerization (CP) of tetraethoxysilane (TEOS) and FRP of (*N*,*N*′-dimethylacrylamide) (DMAAm), which can be achieved by a one-step or a two-step method (Figure 5a). The physically cross-linked silica network is rigid and fragile. In contrast, the covalently cross-linked PDMAAm network is soft and stretchable. When loading the DN ionogel obtained by the two-step process, the fragile network tends to be disrupted to dissipate energy, yielding a high compressive strength of ∼28 MPa. However, the silica network is less reversible at room temperature and requires annealing (*i*.*e*., 100 °C) to reform.

It is also possible to form inorganic–organic DN ionogels in a single step by simultaneously triggering the polycondensation of TEOS and the polymerization of DMAAm (Figure 5a). Similar to the previous example, this DN ionogel also uses the silica network as a sacrificial network to enhance the mechanical properties (Figure 5c).^55^ The DN ionogel exhibited a fracture strength of 3.7 MPa with a Young’s modulus of ∼2.5 MPa and can endure a pressure of 80 MPa without damage.^55^ The synthetic strategies discussed above use simple process of CP and FRP (Figure 5d**)**. The materials are dissolved into ILs and then polymerized to form DN ionogels with homopolymer networks. The resultant DN ionogels show excellent compressive properties and their tensile strength are also enhanced compared with previous work.

### 3.2 Toughening by Noncovalent Cross-Links

#### 3.2.1 Polymer Based Noncovalent Cross-Links

In addition to double networks, dynamic bonds (*i*.*e*., noncovalent interactions, such as ionic and hydrogen bonds, electrostatic interactions) can also be introduced into the polymer networks as sacrificial bonds to enhance the toughness.^67,68^ Interestingly, noncovalent bonds are intrinsically reversible so they could endow ionogels with multiple functions, like self-healing, recovery, and shape-memory.^22,23,39,69^

**Figure 6:**
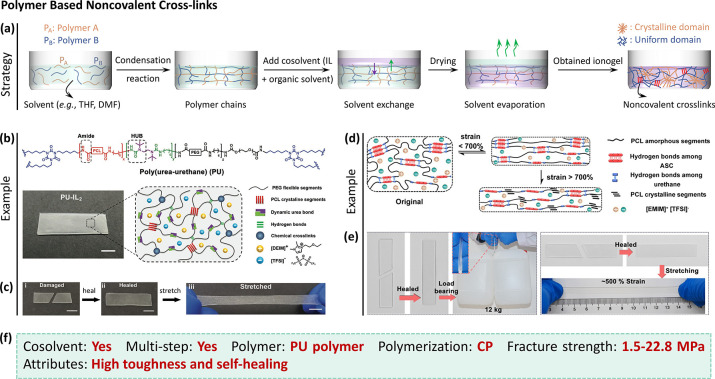
Polymer based noncovalent cross-links toughened ionogels. (a) Procedure of assembling polymer based ionogels. (b) Schematic structure and (c) self-healing property of polyurethane (PU) based ionogel.^39^ Reproduced with permission from ref (39). Copyright 2020 Wiley-VCH. (d) Toughening mechanism and (e) excellent self-healing behavior of PCL based ionogel.^38^ Reproduced from ref (38). Copyright 2022 American Chemical Society. (f) Synthesis features and performance of these tough ionogels.

Poly(urea-urethane) (PU) polymers can be used to produce tough ionogels by solvent exchange because they are difficult to directly dissolve in ILs. Specifically, the synthesized/modified polymers are dissolved in organic solvent (*e*.*g*., tetrahydrofuran (THF) or dimethylformamide (DMF)), which is then replaced by ILs to form ionogels with crystalline domains and noncovalent cross-links (Figure 6a). For instance, a PU based ionogel was synthesized by replacing THF with IL (Figure 6b).^39^ The PU network is covalently cross-linked and consists of poly(ε-caprolactone) (PCL) and poly(ethylene glycol) (PEG) segments. The PCL segment crystallizes in the IL due to their poor compatibility with IL, while the PEG segment is homogeneously dispersed. This example illustrates the importance of choosing an IL with selective solubility with the blocks of the polymer. In addition, the network forms dynamic bonds such as hindered urea bonds between PU chains and hydrogen bonds between the amide groups on PCL. Due to the energy dissipation of these dynamic bonds and the crystalline domains, the PU based ionogel gave a fracture strength of 1.56 MPa. As expected, the dynamic bonds endow the ionogel with self-healing. The healed samples could be stretched to 250% strain (Figure 6c).^39^ However, organic solvents and laborious synthetic chemistry are required.

A similar method has been used to prepare a PCL based PU ionogel.^38^ To introduce dynamic bonds into the PU network, PCL chains were modified with urethane and acylsemicarbazide (ASC). The network can form hydrogen bonds (although the authors do not comment on it, the formation of such bonds suggests that portions of the network are poorly solvated by the IL). As shown in Figure 6d, when stretching the ionogel, energy is dissipated by breaking the hydrogen bonds. At larger strains (> 700% strain), the PCL chains crystallize due to alignment.^38^ Thus, the synergy of these phenomena impart excellent tensile properties to the ionogel (including fracture strength of 22.8 MPa, fracture strain of ∼2100% and toughness of 73.6 kJ m^–2^) and good self-healing capability (Figure 6e).^38^ After elongating to 500% strain, the ionogel can fully recover after 2 h at 25 °C. Yet, for large strain (*i*.*e*., > 700%), heating may be required for fully recovery due to crystallization of PCL chains. The polymer based ionogels have high toughness, fracture strength, and self-healing property (Figure 6f). Yet, the drawback of this approach is that the polymers require synthetic chemistry involving the use of solvent exchange, which produces waste and adds extra processing steps.

#### 3.2.2 Noncovalent Cross-Links Mediated by ILs

**Figure 7:**
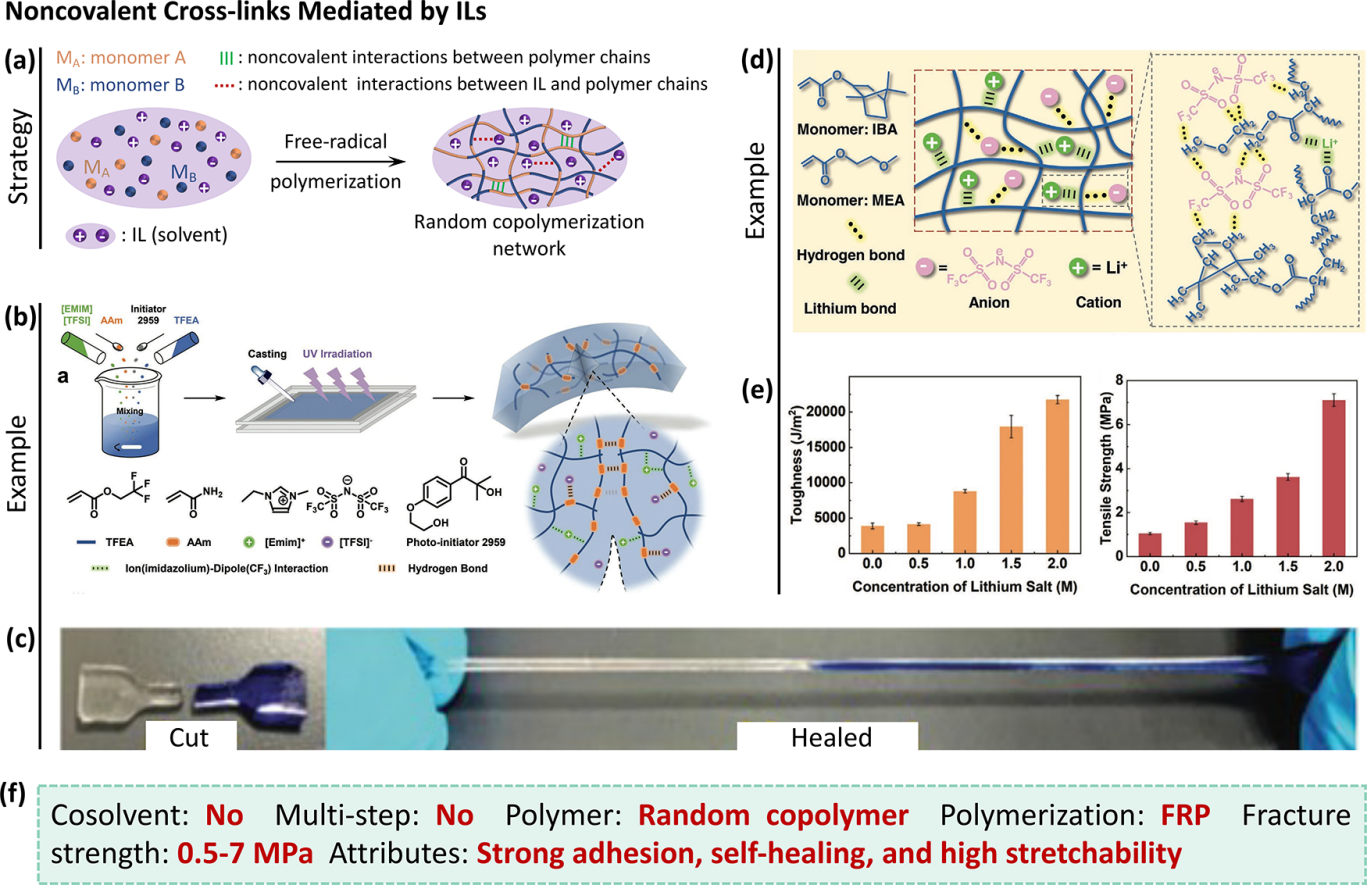
Noncovalent cross-links mediated by ILs. (a) Schematic illustrating the simple procedure for synthesis of tough ionogels. (b) One-pot strategy to achieve highly stretchable and self-healing P(AAm-*co*-TFEA) ionogel via random copolymerization.^58^ (c) P(AAm-*co*-TFEA) ionogel shows excellent self-healing.^58^ Panels (b) and (c) were reproduced with permission from ref (58). Copyright 2021 Wiley-VCH. (d) Hydrogen bonds and ion-dipole interactions form in situ in the P(IBA-*co*-MEA) elastomer network using a one-step method.^51^ (e) Toughness and fracture strength of the P(IBA-*co*-MEA) elastomer monotonically increase as Li salt concentration due to the number of dynamic bonds increasing.^51^ Panels (d) and (e) were reproduced with permission from ref (51). Copyright 2021 Wiley-VCH. (f) Summary of the synthetic process and properties of the ionogels.

The aforementioned strategies dissipate energy via noncovalent bonds between the polymer chains.^38,39^ Yet, physical interactions between ILs and polymer network can also toughen ionogels.^7,70−72^ Importantly, ionogels with noncovalent cross-links can be achieved by simply polymerizing monomers in ILs (Figure 7a). The physical interactions form *in situ* between adjacent polymer chains or between ILs and polymer chains that act as dynamic cross-links. For example, a noncovalently cross-linked ionogel was produced using a one-pot process by copolymerizing acrylamide (AAm) and 2,2,2-trifluoroethyl acrylate (TFEA) monomers in ionic liquid of 1-ethyl-3-methylimidazolium bis(trifluoromethanesulfonyl)imide ([EMIM][TFSI]) (Figure 7b).^58^ Notably, both the TFEA monomer and the TFSI anions in the ionic liquid are fluorinated and the amide group hanging on AAm is a good hydrogen bond donor and acceptor. Thus, ion-dipole interactions can form between the EMIM cations in ionic liquid and TFEA in the polymer network, due to the high electronegativity of fluorine. Besides AAm in the copolymer chains, hydrogen bonds also form between AAm and TFSI anions in the ionic liquid. These hydrogen bonds and ion-dipole interactions render the ionogel highly stretchable (> 1000% strain) and self-healing (∼100% healing efficiency) (Figure 7c), and provide good compressive properties (*e*.*g*., compressive strength of ∼13 MPa at 90% strain).

Similarly, a tough and self-healing elastomer containing lithium bis(trifluoromethane)sulfonimide salt (LiTFSI, Li salt) was created using a single step by copolymerizing ethylene glycol methyl ether acrylate (MEA) and isobornyl acrylate (IBA) monomers (Figure 7d).^51^ Note that LiTFSI is solid and soluble in the monomers, so this material may be considered an ionic elastomer rather than an ionogel. The cations (*i*.*e*., Li^+^) and anions from LiTFSI could form electrostatic interactions and hydrogen bonds with the polymer network, respectively. These dynamic bonds can break to dissipate energy, resulting in high toughness and self-healing. Importantly, the mechanical performance (*i*.*e*., toughness and fracture strength) of the elastomer were improved monotonically with increased concentration of Li salt due to the increased number of noncovalent interactions (Figure 7e).^51^ The toughness and fracture strength reached maxima of ∼22 000 J m^–2^ and ∼7 MPa, respectively, which are comparable to some tough hydrogels, cartilage, and natural rubber.^1,3,52,53^ This approach is noteworthy for another reason: Whereas the aforementioned strategies to toughen ionogels (in this section) often involve complex synthetic procedures and toxic organic solvents, the single step approach used here is simple.

The tough materials in this section are prepared by a one-step polymerization method (Figure 7f). The noncovalent interactions effectively dissipate energy during deformation yielding good mechanical properties. Additionally, these reversible bonds endow the materials with excellent self-healing and adhesive properties. This simple approach should broaden the applications of ionogel because it is easy for nonchemists to implement.

### 3.3 Phase Separation for Toughening

#### 3.3.1 Block Copolymer Based Phase Separation

Phase separation is another common strategy for toughening gels. Phase separation implies that some portion (or all) of the polymer network is poorly solvated by the ILs and occurs due to the poor compatibility between polymer chains and solvents.^2,19,22^ In phase-separated domains, the noncovalent nature of interactions between polymer chains can impart toughening. Phase separation was initially demonstrated to form hydrogels and has been widely exploited to produce various tough hydrogels.^34,63,73^ For ionogels, phase behavior has been rarely studied, which may be due to the complex interactions between polymers and ILs.^2,61^

**Figure 8:**
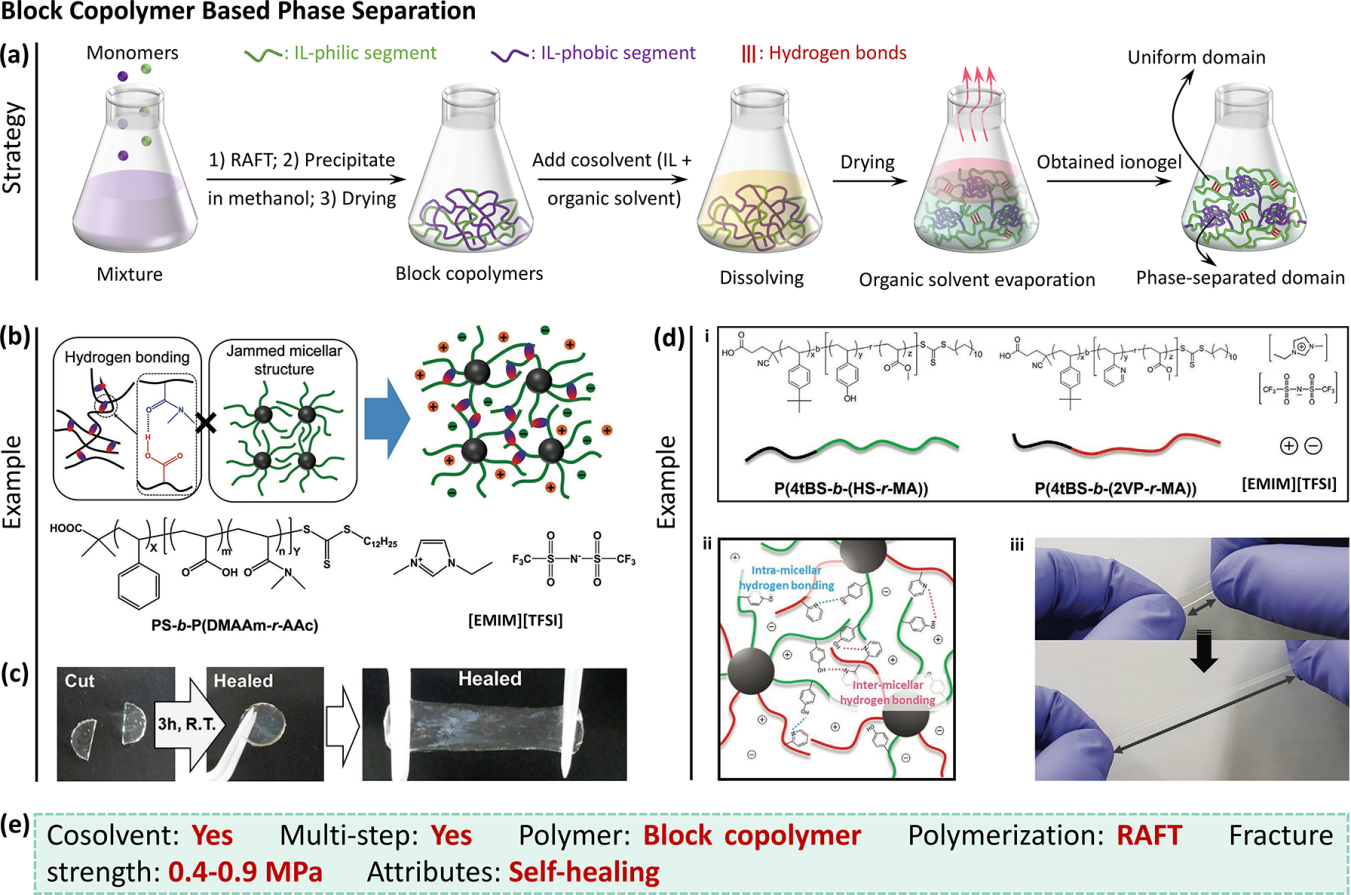
Block copolymer-based phase-separated ionogels. (a) Schematics for the synthetic process. (b) Topological structure of a diblock copolymer, PS-*b*-P(DMAAm-*r*-AAc), based ionogel.^61^ The PS (*i*.*e*., IL-phobic) blocks aggregate to form phase separation, while hydrogen bonds are formed between the solvated P(DMAAm-*r*-AAc) (*i*.*e*., IL-philic) blocks. (c) Hydrogen bonds endow the PS-*b*-P(DMAAm-*r*-AAc) ionogel with good self-healing behavior.^61^ b and c were reproduced with permission from ref (61). Copyright 2018 Wiley-VCH. (d) Two block copolymers form an ionogel with phase-separated micellar clusters and hydrogen bonds, which enable high strain.^74^ Reproduced with permission from ref (74). Copyright 2021 Wiley-VCH. (e) The synthesis features and resultant properties of block copolymer based ionogels.

Phase-separated ionogels can form from block copolymers in which one block has poor solubility in ILs and thus form phase-separated domains (note: we mention block copolymers in section 3.2.1, but those papers do not explicitly mention phase separation so we have not included them in this section). Often the block copolymers are synthesized via reversible addition–fragmentation chain-transfer (RAFT) polymerization followed by precipitation and drying (Figure 8a). Cosolvent (*i*.*e*., a mixture of IL and organic solvent such as THF) is usually used to simultaneously dissolve the block copolymer and form ionogels via solvent evaporation.^62^ This cosolvent approach is necessary since the IL does not fully dissolve the polymer by itself. As the organic solvent evaporates, portion of the block copolymers phase separate to form the gel due to the poor solubility in the IL.

For example, IL ([EMIM][TFSI]) and diblock copolymer, poly(styrene)-*b*-poly(N,N-dimethylacrylamide-*r*-acrylic acid) (PS-*b*-P(DMAAm-*r*-AAc) are dissolved in a cosolvent of methanol and dichloromethane to create an ionogel (Figure 8b).^61^ In the diblock copolymer chains, PS blocks have poor compatibility with the IL. Thus, the PS blocks aggregate to form phase separation by excluding the IL. Meanwhile, hydrogen bonds can form between the P(DMAAm-*r*-AAc) blocks. Hence, the ionogel network consists of hard (phase-separated domains formed by PS block) and soft (hydrogen-bonded P(DMAAm-*r*-AAc) block) phases. The hydrogen bonds dissipate energy by rupturing and enable self-healing by automatically reforming. The ionogels showed excellent self-healing property (*i*.*e*., almost fully healed at room temperature for 3 h) (Figure 8c). But the mechanical performance, such as fracture strength (∼0.3 MPa) and elongation (∼400%), are relatively low.^61^ Likewise, a phase-separated ionogel was created via a block copolymer of poly(methyl methacrylate-*ran*-butyl acrylate) (PMMA-*r*-PBA).^62^ Owing to the low miscibility of PBA blocks in [EMIM][TFSI], phase-separated domains form that remain connected by soft phases of solvated PMMA. The obtained PMMA-*r*-PBA ionogel was stretched to ∼850% with a small fracture strength of ∼0.2 MPa.

Notably, these phase-separated ionogels are designed to achieve self-healing or highly stretchable ionogels. To improve the mechanical performance, two block copolymers containing an IL-phobic block and IL-philic block are used as the gel network (Figure 8d (i)).^74^ The IL-phobic blocks phase separate to form micellar clusters connected by solvated and hydrogen-bonded IL-philic blocks, where the hydrogen bonds act as sacrificial bonds to dissipate energy and toughen the ionogel (Figure 8d (ii)). As shown in Figure 8d (iii), the ionogel could be elongated to ∼7 times its original length.^74^ Yet, the fracture strength was only slightly improved to ∼0.65 MPa, which is much lower than that of phase-separated hydrogels (*e*.*g*., fracture strength: ∼7 MPa).^34,73^

From the above discussion, we know that block copolymers can form by a separate polymerization step (Figure 8e). The synergy of hydrogen bonds (formed between IL-philic segments) and phase behavior (formed by IL-phobic segments) produces ionogel with modest mechanical performance. In general, reversible hydrogen bonds can endow ionogels with self-healing, which may improve stability, lifetime, and application space of ionogels.

#### 3.3.2 Random Copolymer Based Phase Separation

Based on the above discussion, producing ionogels with the best properties often requires significant processing such as multiple reaction steps and/or solvent exchange. While some complicated strategies can achieve tough ionogels with properties similar to tough hydrogels or biological tissues, most approaches do not.

**Figure 9:**
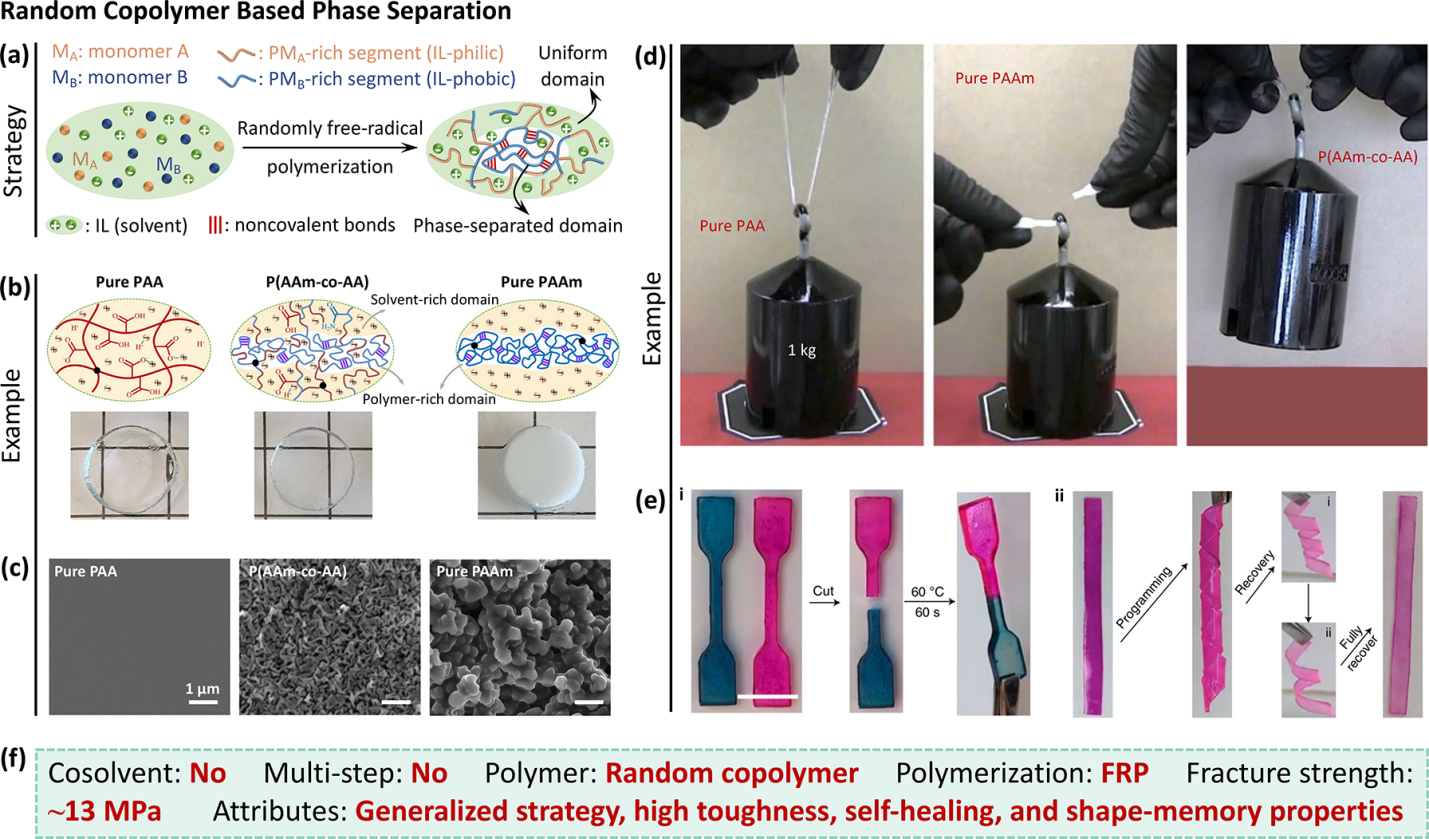
Random copolymer based phase-separated ionogel. (a) One-step strategy for preparation of phase separation toughened ionogels. (b) The toughening mechanism of P(AAm-*co*-AA) ionogel.^22^ That is, hydrogen bonds in the polymer-rich domains are ruptured first to dissipate energy, while the solvent-rich domains deform to stabilize the deformation. (c) SEM images reveal the microstructures of various ionogels.^22^ All the SEM samples were observed without removing EMIES from the polymer networks. (d) High toughness of P(AAm-*co*-AA) ionogel. The copolymer ionogel lifted the 1 kg weight, while the pure PAA and PAAm ionogels failed.^22^ The ionogels are dog-bone shaped with a cross-sectional area of 4 mm^2^. (e) P(AAm-*co*-AA) ionogel exhibits good self-healing (the scale bar is 1 cm) and shape-memory properties due to phase behavior.^22^ (f) Synthetic features and properties of the phase-separated ionogel. Panels (b)–(e): reproduced with permission from ref (22). Copyright 2022 Springer Nature.

Recently, we developed a simple one-step method to create tough ionogels.^22^ The ionogel is toughened by phase separation that occurs *in situ* during polymerization in an IL solvent. The design principle is to use two monomers that start out soluble in the ionic liquid, but one of which phase separates when polymerized (Figure 9a). When the monomers are copolymerized at a certain molar fraction (relative to the total monomer concentration), it will partially phase separate into small domains that toughen the network. The phase-separated domains can be sufficiently small as to produce a transparent ionogel.

In our initial work, we copolymerized acrylamide (AAm) and acrylic acid (AA) monomers in 1-ethyl-3-methylimidazolium ethyl sulfate (EMIES). Note that these are very common and inexpensive monomers. Poly(acrylic acid) (PAA) chains have good compatibility with EMIES forming a homogeneous gel network, while polyacrylamide (PAAm) polymers are insoluble, repelling EMIES molecules to form phase separation (Figure 9b).^22^ Thus, PAA ionogel is transparent and the scanning electron microscope (SEM) image shows a uniform morphology (Figure 9c). In contrast, PAAm ionogel is opaque due to the phase separation. If AAm and AA are randomly copolymerized, two phases form in the P(AAm-*co*-AA) copolymer ionogel: a solvent-rich domain and polymer-rich domain (Figure 9b). The copolymer ionogels are macroscopically transparent over a range of compositions, but SEM images show microphase separation.

Notably, in the polymer-rich domains, hydrogen bonds can form between PAAm chains via amide groups. Hydrogen bonds dissipate energy during loading, while the solvent-rich domains are elastic and distribute the stress. The synergy of these two phases makes the copolymer ionogel ultratough. For example, a small sample of the copolymer ionogel (cross-sectional area: 4 mm^2^) could lift a 1 kg weight, while the pure PAA and PAAm ionogels failed (Figure 9d).^22^ Additionally, the copolymer ionogels exhibited high fracture strength (∼13 MPa), Young’s modulus (∼50 MPa), fracture energy (∼24 000 J m^–2^), and large stretchability (∼600%). These properties outperform most tough hydrogels and ionogels, natural rubber, and cartilage.^35,52,53,65^ Importantly, the synthesis is simple. Polymerization occurs via free-radical initiation, which is achieved by simply adding an initiator to the IL and monomers. The initiator can respond to heat or light. In other words, it is possible for a nonchemist to simply combine the components and shine light to produce tough ionogels–a process that is compatible with 3D printing and other 1-step processes such as coatings.

The phase behavior is temperature dependent. That is, the polymer-rich domains soften (redissolve in IL) under heating (*e*.*g*., 60 °C), and thus, new polymer-rich domains with hydrogen bonds form upon cooling. This reversible process enables healing (Figure 9e).^22^ Similarly, softening of the covalently cross-linked network provides the driving force for chains to recover their original conformation to realize shape-memory. Importantly, this facile one-step strategy is general (Figure 9f). That is, it is not limited to a single set of monomers and ionic liquid. The principle works as long as the polymer chains could partially phase separate in ILs. The ability to form tough gels in a simple manner should broaden the application of these materials.

## 4 Conclusions and Prospects

### 4.1 Brief Perspective

While there are a number of ways to make tough ionogels, they typically have two common features: (1) cross-links–either covalent or noncovalent—to hold the gel together, and (2) secondary interactions such as hydrogen bonds or ionic interactions to help dissipate energy. Compared to hydrogels, toughened ionogels are underexplored. This could partly be due to an insufficient understanding of ILs and the interactions between polymers and ILs.^2,33,61,63^ The lack of understanding is partly a consequence of the enormous diversity of ILs (∼10^18^ species).^9,12,15^ As a result, it is a challenge to achieve tough ionogels only by free-radical polymerization. This may explain why solvent exchange is commonly used to make tough ionogels.

To elaborate on why solvent exchange is utilized: (1) Tough ionogels could be produced based on the principles used for tough hydrogels, which are more developed. As an extreme case, one could imagine making a tough hydrogel and then replacing water with ILs to obtain the ionogels. But this strategy does not always work well because ILs are different from water in physicochemical properties, so the polymers behave differently than they do in water. Thus, the resultant ionogels may not be tough. (2) Due to this, polymers such as PU and block copolymers are used to produce tough ionogels. These polymers have IL-phobic segments, which have to be dissolved by organic solvents for the sake of processing (*i*.*e*., dissolving and casting films). After removing the organic solvent, the obtained ionogels may be toughened by phase behavior (due to IL-phobic segments) and noncovalent interactions such as hydrogen bonds. Compared with (1), (2) produces tough ionogels more efficiently, which explains the widespread use of solvent exchange.

When solvent exchange is involved, the synthetic process is complicated.^39,40,50,75^ For example, researchers prepared DN hydrogels first and then replaced water by ILs to yield tough ionogels.^50^ This approach is laborious and costly. Nonetheless, simplified strategies have been reported, but the improvements in mechanical properties are unsatisfactory as they are still far lower than that of tough hydrogels.^51,58^ Therefore, phase separation via *in situ* polymerization may be a promising way to fabricate tough ionogels to avoid solvent exchange and multistep synthesis.

Additionally, there are other ways to toughen ionogels. For example, few studies have been reports on the addition of fillers (*e*.*g*., carbon nanotubes, clay, MOFs, TiO_2_, and Fe_3_O_4_ nanoparticles), which is also an effective way to introduce sacrificial bonds to toughen gels.^23,54,76^ More attention is needed in these areas to create tough ionogels with self-healing, self-recovery, and shape-memory properties and thus broaden the applications of ionogels.

### 4.2 Overview

Ionogels form a new class of soft materials that are in their infancy. Relative to hydrogels, they are attractive because ionogels do not evaporate, they have ionic conductivity and exceptional thermal and electrochemical stability. Yet, most ionogels are too soft or brittle to be useful. This Perspective has highlighted efforts to create tough ionogels in terms of synthetic methods and toughening mechanism.

ILs are functional solvents in which the properties of both cation and anion components can be independently modified. Thus, about 10^18^ ILs are thought to be possible. This is both a blessing and a curse. It is a blessing because it can enable a wide variety of physicochemical properties and thus diverse ionogels. Yet it is a curse because the parameter space is enormous.

A greater understanding of interactions between ILs and polymers would accelerate the development of ionogels. While dissipation-induced toughening is a significant guiding principle for achieving tough gels, it has only started to be applied in ionogels. The principle is to introduce weak or dynamic bonds between polymer chains (or between polymer chains mediated by ionic liquids) in ionogel networks. These bonds serve as sacrificial bonds to dissipate a large amount of energy during deformation, thereby toughening the gels. The combination of this principle and the sufficient understanding of interactions between ILs and polymers can help to design tough ionogels.

We hope this Perspective will draw attention to opportunities to improve upon the synthesis, understanding, and properties of ionogels. With the achievements, studies on the interactions between polymers and ILs and properties of ionogels would be more comprehensive. In turn, this understanding will increase the applications of ionogels.

### 4.3 Synthesis and IL-Polymer Thermodynamic Interactions

To date, most synthetic approaches to form tough ionogels require solvent exchange, which is a multistep and laborious process that generates waste. It would be desirable to form the ionogels by polymerizing directly in ILs—including simple polymerization mechanisms, such as free-radical polymerization—but doing so requires choosing an ionic liquid with appropriate interactions with both the monomer and polymer. Because ILs are diverse and have significant difference in physicochemical properties, it is often difficult for a synthetic chemist or engineer to know which IL to choose. Consequently, polymerization within IL has not been exploited as much as other approaches. Since ionogels are different from hydrogels, one cannot directly apply the working principles of monomers or polymers established in water to ILs. The key is to understand the physicochemical properties of ILs and the interactions between ILs and polymers. In addition to solubility pre- and postpolymerization, the IL can affect the polymer-IL interactions. For example, efforts have demonstrated that proper selection of ILs (such as fluorinated ILs) could form noncovalent interactions between ILs and polymer chains (*i*.*e*., PAAm), which can act as sacrificial bonds to toughen ionogels.^51,58^

Only by fully studying the properties of ILs and the interactions between polymers and ILs can we tailor ionogels and optimize the synthetic strategies. For instance, we were able to create tough ionogels in a single step by knowing that AAm monomer, AA monomer, and PAA polymer are soluble in EMIES, but PAAm polymer is insoluble (see section 3.3.2).^22^ Hence, the copolymer chains contain segments having good and poor compatibility with EMIES after randomly copolymerizing AAm and AA. The segments with poor solubility phase separate to form noncovalent bonds between polymer chains, thereby improving the mechanical properties of the resulting ionogel. Importantly, this one-step strategy is applicable to other monomers and ILs as long as the polymeric segments could phase separate in the ILs. This is of great significance for simplifying the synthesis of various tough ionogels and further studying their applications.

### 4.4 Recovery

Recovery is the ability of gels to recover to their original mechanical properties and shape after deformation. Recovery requires the network to have some elasticity. Yet, elastic materials by themselves require some secondary interactions to provide toughness.^3,33^ These bonds can either be intrinsically reversible (noncovalent) or irreversible (breaking covalent bonds permanently). When irreversible bonds rupture during deformation, the gel may only recover partially since irreversible bonds cannot reform. If only noncovalent or dynamic bonds break during deformation, the gel may recover fully. The recovery ability can be improved or sped up by external stimuli such as heating. For example, a tough ionogel with phase-separated domains containing hydrogen bonds could almost fully recover by heating it at 60 °C for 2 h after initially elongating it to 500% strain.^22^ Recovery is important, especially for large deformation applications. Yet, the current efforts toward tough ionogels has focuses on the synthetic methods and mechanical properties. Attention needs to be paid to improve the recovery properties of ionogels. One route is to avoid irreversible bonds and instead using reversible interactions (*e*.*g*., hydrogen bonds, electrostatic interactions, dynamic disulfide bonds).

### 4.5 Conductivity

Ionogels inherit the good conductivity of ILs. Yet, ionogel conductivity varies by the morphology of the polymer network, which affects the movement of bulky organic ions. For instance, the conductivity of the PAMPS DN ionogels decreased from 1.7 to 0.6 S m^–1^ as the porous structure became denser.^50^ In contrast, when ILs contain small ions such as Li^+^, the ions movement is less affected by the polymer network. Thus, ionogels with Li^+^ as the cation have great potential in solid-state electrolytes in Li-ion batteries.^5,9^ These results indicate that the conductive property of ionogels can be tuned by the morphology of the gel network or by choosing ILs. Yet, their conductive behavior under different temperatures has been rarely studied. Combining the high thermal stability and low volatility of ionogels may enable new application areas such as high temperature sensors.

### 4.6 Potential Applications

Creating ionogels with desirable mechanical properties via simple processes or chemistries is highly attractive because it can broaden their applications in promising fields like actuators and sensors, soft robots, and energy storage devices. For example, solid electrolytes in Li-ion batteries require a large modulus of ∼60 MPa to suppress the growth of Li dendrites, a safety issue leading to short circuits.^5^ Some applications can be further enabled by using ionogels that can be 3D printed, which is compatible with ionogels that can be photopolymerized in a single exposure to light. However, this class of materials has been overlooked for 3D printing.

In addition to the above fields, the ionic conductivity of ionogels makes them promising in soft/wearable electronics and energy harvesters (*e*.*g*., convert mechanical energy into electrical energy). One unique application may be in outer space; since ionogels have almost zero vapor pressure and a wide temperature working window, their potential for applications in outer space can be tapped.

## References

[ref1] Gong J. P. Why are double network hydrogels so tough?. Soft Matter 2010, 6 (12), 2583–2590. 10.1039/b924290b.

[ref2] Ueki T.; Watanabe M. Polymers in ionic liquids: dawn of neoteric solvents and innovative materials. Bull. Chem. Soc. Jpn. 2012, 85 (1), 33–50. 10.1246/bcsj.20110225.

[ref3] Sun J.-Y.; Zhao X.; Illeperuma W. R.; Chaudhuri O.; Oh K. H.; Mooney D. J.; Vlassak J. J.; Suo Z. Highly stretchable and tough hydrogels. Nature 2012, 489 (7414), 133–136. 10.1038/nature11409. 22955625 PMC3642868

[ref4] Shi P.; Wang Y.; Wan K.; Zhang C.; Liu T. A Waterproof Ion-Conducting Fluorinated Elastomer with 6000% Stretchability, Superior Ionic Conductivity, and Harsh Environment Tolerance. Adv. Funct. Mater. 2022, 32 (22), 2112293 10.1002/adfm.202112293.

[ref5] Chen N.; Zhang H.; Li L.; Chen R.; Guo S. Ionogel electrolytes for high-performance lithium batteries: A review. Adv. Energy Mater. 2018, 8 (12), 1702675 10.1002/aenm.201702675.

[ref6] Shi L.; Jia K.; Gao Y.; Yang H.; Ma Y.; Lu S.; Gao G.; Bu H.; Lu T.; Ding S. Highly stretchable and transparent ionic conductor with novel hydrophobicity and extreme-temperature tolerance. Research 2020, 2020, 2505619 10.34133/2020/2505619. 33029586 PMC7520821

[ref7] Cao Z.; Liu H.; Jiang L. Transparent, mechanically robust, and ultrastable ionogels enabled by hydrogen bonding between elastomers and ionic liquids. Mater. Horiz. 2020, 7 (3), 912–918. 10.1039/C9MH01699F.

[ref8] Osada I.; de Vries H.; Scrosati B.; Passerini S. Ionic-liquid-based polymer electrolytes for battery applications. Angew. Chem. In. Ed. 2016, 55 (2), 500–513. 10.1002/anie.201504971. 26783056

[ref9] Luo Z.; Li W.; Yan J.; Sun J. Roles of Ionic Liquids in Adjusting Nature of Ionogels: A Mini Review. Adv. Funct. Mater. 2022, 32, 2203988 10.1002/adfm.202203988.

[ref10] Wang Y.; Kalytchuk S.; Zhang Y.; Shi H.; Kershaw S. V.; Rogach A. L. Thickness-dependent full-color emission tunability in a flexible carbon dot ionogel. J. Phys. Chem. Lett. 2014, 5 (8), 1412–1420. 10.1021/jz5005335. 26269987

[ref11] MacFarlane D. R.; Forsyth M.; Howlett P. C.; Kar M.; Passerini S.; Pringle J. M.; Ohno H.; Watanabe M.; Yan F.; Zheng W.; Zhang S.; Zhang J. Ionic liquids and their solid-state analogues as materials for energy generation and storage. Nat. Rev. Mater. 2016, 1 (2), 1–15. 10.1038/natrevmats.2015.5.

[ref12] Marr P. C.; Marr A. C. Ionic liquid gel materials: applications in green and sustainable chemistry. Green Chem. 2016, 18 (1), 105–128. 10.1039/C5GC02277K.

[ref13] Qian W.; Texter J.; Yan F. Frontiers in poly (ionic liquid) s: syntheses and applications. Chem. Soc. Rev. 2017, 46 (4), 1124–1159. 10.1039/C6CS00620E. 28180218

[ref14] Correia D. M.; Fernandes L. C.; Martins P. M.; García-Astrain C.; Costa C. M.; Reguera J.; Lanceros-Méndez S. Ionic liquid–polymer composites: A new platform for multifunctional applications. Adv. Funct. Mater. 2020, 30 (24), 1909736 10.1002/adfm.201909736.

[ref15] Rogers R. D.; Seddon K. R. Ionic liquids--solvents of the future?. Science 2003, 302 (5646), 792–793. 10.1126/science.1090313. 14593156

[ref16] Dai X.; Zhang Y.; Gao L.; Bai T.; Wang W.; Cui Y.; Liu W. A Mechanically Strong, Highly Stable, Thermoplastic, and self-healable supramolecular polymer hydrogel. Adv. Mater. 2015, 27 (23), 3566–3571. 10.1002/adma.201500534. 25946310

[ref17] Kim Y. S.; Liu M.; Ishida Y.; Ebina Y.; Osada M.; Sasaki T.; Hikima T.; Takata M.; Aida T. Thermoresponsive actuation enabled by permittivity switching in an electrostatically anisotropic hydrogel. Nat. Mater. 2015, 14 (10), 1002–1007. 10.1038/nmat4363. 26259107

[ref18] Lodge T. P.; Ueki T. Mechanically tunable, readily processable ion gels by self-assembly of block copolymers in ionic liquids. Acc. Chem. Res. 2016, 49 (10), 2107–2114. 10.1021/acs.accounts.6b00308. 27704769

[ref19] Ueki T.; Watanabe M.; Lodge T. P. Doubly thermosensitive self-assembly of diblock copolymers in ionic liquids. Macromolecules 2009, 42 (4), 1315–1320. 10.1021/ma802443b.

[ref20] Li W.; Li L.; Zheng S.; Liu Z.; Zou X.; Sun Z.; Guo J.; Yan F. Recyclable, Healable, and Tough Ionogels Insensitive to Crack Propagation. Adv. Mater. 2022, 34, 2203049 10.1002/adma.202203049. 35522456

[ref21] Xia Q.; Li W.; Zou X.; Zheng S.; Liu Z.; Li L.; Yan F. Metal-Organic Frameworks (MOFs) Facilitated Highly Stretchable, and Fatigue-Resistant Ionogels for Recyclable Sensors. Mater. Horiz. 2022, 9, 2881–2892. 10.1039/D2MH00880G. 36097959

[ref22] Wang M.; Zhang P.; Shamsi M.; Thelen J. L.; Qian W.; Truong V. K.; Ma J.; Hu J.; Dickey M. D. Tough and stretchable ionogels by in situ phase separation. Nat. Mater. 2022, 21 (3), 359–365. 10.1038/s41563-022-01195-4. 35190655

[ref23] Zhang L. M.; He Y.; Cheng S.; Sheng H.; Dai K.; Zheng W. J.; Wang M. X.; Chen Z. S.; Chen Y. M.; Suo Z. Self-healing, adhesive, and highly stretchable ionogel as a strain sensor for extremely large deformation. Small 2019, 15 (21), 1804651 10.1002/smll.201804651. 30990971

[ref24] Xu J.; Wang H.; Du X.; Cheng X.; Du Z.; Wang H. Self-healing, anti-freezing and highly stretchable polyurethane ionogel as ionic skin for wireless strain sensing. Chem. Eng. J. 2021, 426, 130724 10.1016/j.cej.2021.130724.

[ref25] Ren Y.; Liu Z.; Jin G.; Yang M.; Shao Y.; Li W.; Wu Y.; Liu L.; Yan F. Electric-Field-Induced Gradient Ionogels for Highly Sensitive, Broad-Range-Response, and Freeze/Heat-Resistant Ionic Fingers. Adv. Mater. 2021, 33 (12), 2008486 10.1002/adma.202008486. 33576082

[ref26] Song X.; Wang C.; Chen J.; Xin S.; Yuan D.; Wang Y.; Dong K.; Yang L.; Wang G.; Zhang H.; Zhang S. Unraveling the Synergistic Coupling Mechanism of Li^+^ Transport in an “Ionogel-in-Ceramic” Hybrid Solid Electrolyte for Rechargeable Lithium Metal Battery. Adv. Funct. Mater. 2022, 32 (10), 2108706 10.1002/adfm.202108706.

[ref27] Shen Z.; Zhu X.; Majidi C.; Gu G. Cutaneous ionogel mechanoreceptors for soft machines, physiological sensing, and amputee prostheses. Adv. Mater. 2021, 33 (38), 2102069 10.1002/adma.202102069. 34337793

[ref28] Sun Y.; Wang Y.; Liu Y.; Wu S.; Zhang S.; Niu W. Biomimetic Chromotropic Photonic-Ionic Skin with Robust Resilience, Adhesion, and Stability. Ad. Funct. Mater. 2022, 32, 2204467 10.1002/adfm.202204467.

[ref29] Lu B.; Yuk H.; Lin S.; Jian N.; Qu K.; Xu J.; Zhao X. Pure PEDOT:PSS hydrogels. Nat. Commun. 2019, 10 (1), 1043 10.1038/s41467-019-09003-5. 30837483 PMC6401010

[ref30] Liu X.; Liu J.; Lin S.; Zhao X. Hydrogel machines. Mater. Today 2020, 36, 102–124. 10.1016/j.mattod.2019.12.026.

[ref31] Sun J.; Li R.; Lu G.; Yuan Y.; Zhu X.; Nie J. A facile strategy for fabricating multifunctional ionogel based electronic skin. J. Mater. Chem. C 2020, 8 (25), 8368–8373. 10.1039/D0TC01057J.

[ref32] Zhang L.; Jia K.; Wang J.; Zhao J.; Tang J.; Hu J. Stretchable and transparent ionogel-based heaters. Mater. Horiz. 2022, 9, 1911–1920. 10.1039/D1MH01775F. 35532948

[ref33] Zhao X. Multi-scale multi-mechanism design of tough hydrogels: building dissipation into stretchy networks. Soft Matter 2014, 10 (5), 672–687. 10.1039/C3SM52272E. 24834901 PMC4040255

[ref34] Sato K.; Nakajima T.; Hisamatsu T.; Nonoyama T.; Kurokawa T.; Gong J. P. Phase-separation-induced anomalous stiffening, toughening, and self-healing of polyacrylamide gels. Adv. Mater. 2015, 27 (43), 6990–6998. 10.1002/adma.201502967. 26425825

[ref35] Wang Y. J.; Zhang X. N.; Song Y.; Zhao Y.; Chen L.; Su F.; Li L.; Wu Z. L.; Zheng Q. Ultrastiff and tough supramolecular hydrogels with a dense and robust hydrogen bond network. Chem. Mater. 2019, 31 (4), 1430–1440. 10.1021/acs.chemmater.8b05262.

[ref36] Sun T. L.; Kurokawa T.; Kuroda S.; Ihsan A. B.; Akasaki T.; Sato K.; Haque M.; Nakajima T.; Gong J. P. Physical hydrogels composed of polyampholytes demonstrate high toughness and viscoelasticity. Nat. Mater. 2013, 12 (10), 932–937. 10.1038/nmat3713. 23892784

[ref37] Hu X.; Vatankhah-Varnoosfaderani M.; Zhou J.; Li Q.; Sheiko S. S. Weak hydrogen bonding enables hard, strong, tough, and elastic hydrogels. Adv. Mater. 2015, 27 (43), 6899–6905. 10.1002/adma.201503724. 26436409

[ref38] Guan T.; Wang X.; Zhu Y.-L.; Qian L.; Lu Z.; Men Y.; Li J.; Wang Y.; Sun J. Mechanically Robust Skin-like Poly (urethane-urea) Elastomers Cross-Linked with Hydrogen-Bond Arrays and Their Application as High-Performance Ultrastretchable Conductors. Macromolecules 2022, 55 (13), 5816–5825. 10.1021/acs.macromol.2c00492.

[ref39] Li T.; Wang Y.; Li S.; Liu X.; Sun J. Mechanically robust, elastic, and healable ionogels for highly sensitive ultra-durable ionic skins. Adv. Mater. 2020, 32 (32), 2002706 10.1002/adma.202002706. 32589326

[ref40] Ren Y.; Guo J.; Liu Z.; Sun Z.; Wu Y.; Liu L.; Yan F. Ionic liquid–based click-ionogels. Sci. Adv. 2019, 5 (8), eaax0648 10.1126/sciadv.aax0648. 31467977 PMC6707778

[ref41] Tamura S.; Ueki T.; Ueno K.; Kodama K.; Watanabe M. Thermosensitive self-assembly of diblock copolymers with lower critical micellization temperatures in an ionic liquid. Macromolecules 2009, 42 (16), 6239–6244. 10.1021/ma900922d.

[ref42] Gayet F.; Viau L.; Leroux F.; Monge S.; Robin J.-J.; Vioux A. Polymer nanocomposite ionogels, high-performance electrolyte membranes. J. Mater. Chem. 2010, 20 (42), 9456–9462. 10.1039/c000033g.

[ref43] Ribot J. C.; Guerrero-Sanchez C.; Hoogenboom R.; Schubert U. S. Thermoreversible ionogels with tunable properties via aqueous gelation of an amphiphilic quaternary ammonium oligoether-based ionic liquid. J. Mater. Chem. 2010, 20 (38), 8279–8284. 10.1039/c0jm02061c.

[ref44] Akagi Y.; Sakurai H.; Gong J. P.; Chung U.-i.; Sakai T. Fracture energy of polymer gels with controlled network structures. J. Chem. Phys. 2013, 139 (14), 144905 10.1063/1.4823834. 24116644

[ref45] Watanabe M.; Yamada S.-I.; Sanui K.; Ogata N. High ionic conductivity of new polymer electrolytes consisting of polypyridinium, pyridinium and aluminium chloride. J. Chem. Soc., Chem. Commun. 1993, (11), 929–931. 10.1039/c39930000929.

[ref46] He Y.; Lodge T. P. Thermoreversible ion gels with tunable melting temperatures from triblock and pentablock copolymers. Macromolecules 2008, 41 (1), 167–174. 10.1021/ma702014z.

[ref47] Kawauchi T.; Kumaki J.; Okoshi K.; Yashima E. Stereocomplex formation of isotactic and syndiotactic poly (methyl methacrylate) s in ionic liquids leading to thermoreversible ion gels. Macromolecules 2005, 38 (22), 9155–9160. 10.1021/ma0517594.

[ref48] Gayet F.; Viau L.; Leroux F.; Mabille F.; Monge S.; Robin J.-J.; Vioux A. Unique combination of mechanical strength, thermal stability, and high ion conduction in PMMA–Silica nanocomposites containing high loadings of ionic liquid. Chem. Mater. 2009, 21 (23), 5575–5577. 10.1021/cm9027918.

[ref49] Liu X.; He B.; Wang Z.; Tang H.; Su T.; Wang Q. Tough nanocomposite ionogel-based actuator exhibits robust performance. Sci. Rep. 2014, 4 (1), 6673 10.1038/srep06673. 25327414 PMC4202203

[ref50] Ding Y.; Zhang J.; Chang L.; Zhang X.; Liu H.; Jiang L. Preparation of high-performance ionogels with excellent transparency, good mechanical strength, and high conductivity. Adv. Mater. 2017, 29 (47), 1704253 10.1002/adma.201704253. 29083496

[ref51] Yiming B.; Han Y.; Han Z.; Zhang X.; Li Y.; Lian W.; Zhang M.; Yin J.; Sun T.; Wu Z.; Li T.; Fu J.; Jia Z.; Qu S. A Mechanically Robust and Versatile Liquid-Free Ionic Conductive Elastomer. Adv. Mater. 2021, 33 (11), 2006111 10.1002/adma.202006111. 33576145

[ref52] Corkhill P.; Trevett A.; Tighe B. The potential of hydrogels as synthetic articular cartilage. Proc. Inst. Mech. Eng. H 1990, 204 (3), 147–155. 10.1243/PIME_PROC_1990_204_249_02. 2133781

[ref53] Asaletha R.; Kumaran M.; Thomas S. Thermoplastic elastomers from blends of polystyrene and natural rubber: morphology and mechanical properties. Eur. Polym. J. 1999, 35 (2), 253–271. 10.1016/S0014-3057(98)00115-3.

[ref54] Kamio E.; Yasui T.; Iida Y.; Gong J. P.; Matsuyama H. Inorganic/Organic Double-Network Gels Containing Ionic Liquids. Adv. Mater. 2017, 29 (47), 1704118 10.1002/adma.201704118. 29114950

[ref55] Yu L.; Guo S.; Lu Y.; Li Y.; Lan X.; Wu D.; Li R.; Wu S.; Hu X. Highly tough, Li-metal compatible organic–inorganic double-network solvate ionogel. Adv. Energy Mater. 2019, 9 (22), 1900257 10.1002/aenm.201900257.

[ref56] Chen B.; Lu J. J.; Yang C. H.; Yang J. H.; Zhou J.; Chen Y. M.; Suo Z. Highly stretchable and transparent ionogels as nonvolatile conductors for dielectric elastomer transducers. ACS Appl. Mater. Interfaces 2014, 6 (10), 7840–7845. 10.1021/am501130t. 24758275

[ref57] Lan J.; Li Y.; Yan B.; Yin C.; Ran R.; Shi L.-Y. Transparent stretchable dual-network ionogel with temperature tolerance for high-performance flexible strain sensors. ACS Appl. Mater. Interfaces 2020, 12 (33), 37597–37606. 10.1021/acsami.0c10495. 32700894

[ref58] Xu L.; Huang Z.; Deng Z.; Du Z.; Sun T. L.; Guo Z. H.; Yue K. A Transparent, Highly Stretchable, Solvent-Resistant, Recyclable Multifunctional Ionogel with Underwater Self-Healing and Adhesion for Reliable Strain Sensors. Adv. Mater. 2021, 33 (51), 2105306 10.1002/adma.202105306. 34647370

[ref59] Yu Z.; Wu P. A highly transparent ionogel with strength enhancement ability for robust bonding in an aquatic environment. Mater. Horiz. 2021, 8 (7), 2057–2064. 10.1039/D1MH00461A. 34846483

[ref60] Cao Z.; Liu H.; Jiang L. Hydrogen-bonding-driven tough ionogels containing spiropyran-functionalized ionic liquids. ACS Appl. Polym. Mater. 2020, 2 (6), 2359–2365. 10.1021/acsapm.0c00297.

[ref61] Tamate R.; Hashimoto K.; Horii T.; Hirasawa M.; Li X.; Shibayama M.; Watanabe M. Self-healing micellar ion gels based on multiple hydrogen bonding. Adv. Mater. 2018, 30 (36), 1802792 10.1002/adma.201802792. 30066342

[ref62] Kim Y. M.; Moon H. C. Ionoskins: nonvolatile, highly transparent, ultrastretchable ionic sensory platforms for wearable electronics. Adv. Funct. Mater. 2020, 30 (4), 1907290 10.1002/adfm.201907290.

[ref63] Zhang H. J.; Sun T. L.; Zhang A. K.; Ikura Y.; Nakajima T.; Nonoyama T.; Kurokawa T.; Ito O.; Ishitobi H.; Gong J. P. Tough physical double-network hydrogels based on amphiphilic triblock copolymers. Adv. Mater. 2016, 28 (24), 4884–4890. 10.1002/adma.201600466. 27117393

[ref64] Liu X.; Wen Z.; Wu D.; Wang H.; Yang J.; Wang Q. Tough BMIMCl-based ionogels exhibiting excellent and adjustable performance in high-temperature supercapacitors. J. Mater. Chem. A 2014, 2 (30), 11569–11573. 10.1039/C4TA01944J.

[ref65] Gong J. P.; Katsuyama Y.; Kurokawa T.; Osada Y. Double-network hydrogels with extremely high mechanical strength. Adv. Mater. 2003, 15 (14), 1155–1158. 10.1002/adma.200304907.

[ref66] Chen N.; Dai Y.; Xing Y.; Wang L.; Guo C.; Chen R.; Guo S.; Wu F. Biomimetic ant-nest ionogel electrolyte boosts the performance of dendrite-free lithium batteries. Energy Environ. Sci. 2017, 10 (7), 1660–1667. 10.1039/C7EE00988G.

[ref67] Qiu W.; Chen G.; Zhu H.; Zhang Q.; Zhu S. Enhanced stretchability and robustness towards flexible ionotronics via double-network structure and ion-dipole interactions. Chem. Eng. J. 2022, 434, 134752 10.1016/j.cej.2022.134752.

[ref68] Weng D.; Xu F.; Li X.; Li S.; Li Y.; Sun J. Polymeric complex-based transparent and healable ionogels with high mechanical strength and ionic conductivity as reliable strain sensors. ACS Appl. Mater. Interfaces 2020, 12 (51), 57477–57485. 10.1021/acsami.0c18832. 33306340

[ref69] Kang S.; Park M. J. Tailoring intermolecular interactions in ion gels with rationally designed phosphonic acid polymers. Polym. Chem. 2022, 13, 4372–4383. 10.1039/D2PY00646D.

[ref70] Wang Y.; Sun S.; Wu P. Adaptive Ionogel Paint from Room-Temperature Autonomous Polymerization of α-Thioctic Acid for Stretchable and Healable Electronics. Adv. Energy Mater. 2021, 31 (24), 2101494 10.1002/adfm.202101494.

[ref71] Yu Z.; Wu P. Underwater communication and optical camouflage ionogels. Adv. Mater. 2021, 33 (24), 2008479 10.1002/adma.202008479. 33955597

[ref72] Li W.; Li L.; Zheng S.; Liu Z.; Zou X.; Sun Z.; Guo J.; Yan F. Recyclable, Healable, and Tough Ionogels Insensitive to Crack Propagation. Adv. Mater. 2022, 34, 2203049 10.1002/adma.202203049. 35522456

[ref73] Nonoyama T.; Lee Y. W.; Ota K.; Fujioka K.; Hong W.; Gong J. P. Instant thermal switching from soft hydrogel to rigid plastics inspired by thermophile proteins. Adv. Mater. 2020, 32 (4), 1905878 10.1002/adma.201905878. 31736142

[ref74] Cho K. G.; An S.; Cho D. H.; Kim J. H.; Nam J.; Kim M.; Lee K. H. Block Copolymer-Based Supramolecular Ionogels for Accurate On-Skin Motion Monitoring. Adv. Funct. Mater. 2021, 31 (36), 2102386 10.1002/adfm.202102386.

[ref75] Chen C.; Ying W. B.; Li J.; Kong Z.; Li F.; Hu H.; Tian Y.; Kim D. H.; Zhang R.; Zhu J. A Self-Healing and Ionic Liquid Affiliative Polyurethane toward a Piezo 2 Protein Inspired Ionic Skin. Adv. Funct. Mater. 2022, 32 (4), 2106341 10.1002/adfm.202106341.

[ref76] Liu X.; Taiwo O. O.; Yin C.; Ouyang M.; Chowdhury R.; Wang B.; Wang H.; Wu B.; Brandon N. P.; Wang Q.; Cooper S. J. Aligned ionogel electrolytes for high-temperature supercapacitors. Adv. Sci. 2019, 6 (5), 1801337 10.1002/advs.201801337. PMC6402534 30886792

